# Supplementary material for: Identification of photoperiod-induced specific miRNAs in the adrenal glands of Sunite sheep (Ovis aries)
Source: Front Vet Sci. 2022 Jul 22;9:888207. doi: 10.3389/fvets.2022.888207 (PMC9354845; doi:10.3389/fvets.2022.888207)
Supplement: Supplementary file 1 [file Data_Sheet_1.ZIP › Supplementary file/miRDeep2 file.zip!/miRDeep2 file/Novel_Structure/LP42A3/Novel_28.pdf]

Provisional ID	:	NC_019471.2_16197
Score total	:	2.4
Score for star read(s)	:	-1.3
Score for read counts	:	0
Score for mfe	:	2.1
Score for randfold	:	1.6
Score for cons. seed	:	
Total read count	:	65
Mature read count	:	64
Loop read count	:	0
Star read count	:	1

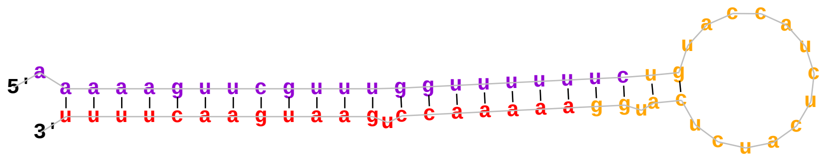

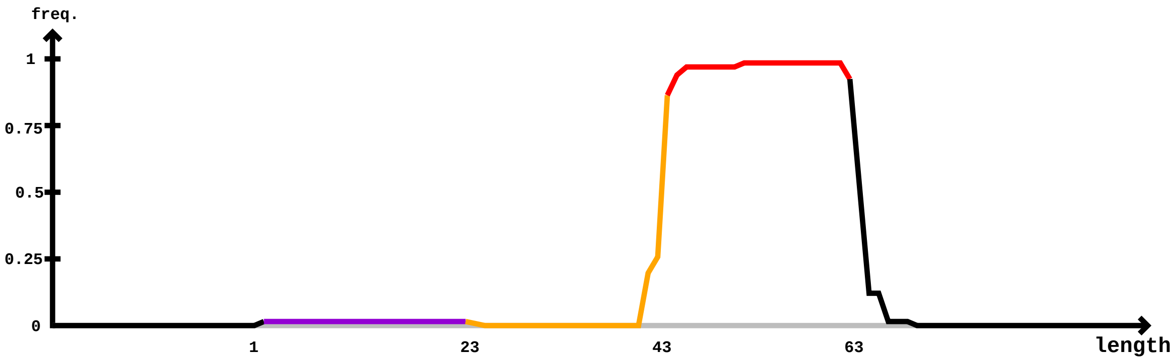


```
                        Star                                     Mature
5'- acuucuguauuggguuggcuaaaaaguucguuugguuuuuucuguaccaucucaucucauggaaaaaccugaaugaacuuuuggccaacccagcagaaauuaucaaggagga   -3'   obs
    acuucuguauuggguuggcuaaaaaguucguuugguuuuuucuguaccaucucaucucauggaaaaaccugaaugaacuuuuggccaacccagcagaaauuaucaaggagga         exp
    ..((((((..((((((((((((((.(((((((((((((((((((.............)).))))))))).))))))))))))))))))))))))))))..............   reads     mm        sample
    ....................aaaaaguucguuugguuuuuuc......................................................................   1         0         seq
    ............................................................ggaaaaaccCgaaugaacuuu...............................   1         1         seq
    ............................................................Agaaaaaccugaaugaacuuu...............................   1         1         seq
    ............................................................Agaaaaaccugaaugaacuuuu..............................   10        1         seq
    ............................................................ggaaaaaccugaaugaacuuuu..............................   1         0         seq
    .............................................................gaaaaaccugaaugaacuuu...............................   1         0         seq
    .............................................................gaaaaaccugaaCgaacuuuu..............................   1         1         seq
    .............................................................gaaaaaccugaaugaacuuuu..............................   2         0         seq
    ..............................................................aaaaaccugaaCgaacuuu...............................   1         1         seq
    ..............................................................aaaaaccugaaCgaacuuuu..............................   7         1         seq
    ..............................................................aaaaaccAgaaugaacuuuu..............................   3         1         seq
    ..............................................................aaaaaccCgaaugaacuuuu..............................   2         1         seq
    ..............................................................aaaaaUcugaaugaacuuuu..............................   23        1         seq
    ..............................................................aaaaaccugaaugaacuuuuC.............................   1         1         seq
    ..............................................................aaaaaccugaaugaacuuuuUgc...........................   3         1         seq
    ...............................................................aaaaccugaaugaacuuuu..............................   2         0         seq
    ...............................................................aaaaccugaaugaacuuuuUgc...........................   2         1         seq
    ...............................................................aaaaUcugaaugaacuuuuggc...........................   1         1         seq
    ................................................................aaaccugaaugaacuuuu..............................   1         0         seq
    ................................................................aaaUcugaaugaacuuuuggc...........................   1         1         seq
    ......................................................................Aaaugaacuuuuggccaa........................   1         1         seq
```
